# Consequences of intraspecific variation in seed dispersal for plant demography, communities, evolution and global change

**DOI:** 10.1093/aobpla/plz016

**Published:** 2019-03-21

**Authors:** Rebecca S Snell, Noelle G Beckman, Evan Fricke, Bette A Loiselle, Carolina S Carvalho, Landon R Jones, Nathanael I Lichti, Nicky Lustenhouwer, Sebastian J Schreiber, Christopher Strickland, Lauren L Sullivan, Brittany R Cavazos, Itamar Giladi, Alan Hastings, Kimberly M Holbrook, Eelke Jongejans, Oleg Kogan, Flavia Montaño-Centellas, Javiera Rudolph, Haldre S Rogers, Rafal Zwolak, Eugene W Schupp

**Affiliations:** 1 Department of Environmental and Plant Biology, Ohio University, Athens, OH, USA; 2 Department of Biology and Ecology Center, Utah State University, Logan, UT, USA; 3 Department of Ecology, Evolution, and Organismal Biology, Iowa State University, Ames, IA, USA; 4 Department of Wildlife Ecology and Conservation, University of Florida, Gainesville, FL, USA; 5 Center for Latin American Studies, University of Florida, Gainsville, FL, USA; 6 Instituto Tecnológico Vale, Belém, Brazil; 7 Department of Forestry and Natural Resources, Purdue University, West Lafayette, IN, USA; 8 Department of Statistics, Purdue University, West Lafayette, IN, USA; 9 Department of Ecology and Evolutionary Biology, University of California, Santa Cruz, CA, USA; 10 Department of Evolution and Ecology and Center for Population Biology, University of California, Davis, CA, USA; 11 Department of Mathematics and Department of Ecology and Evolutionary Biology, University of Tennessee, Knoxville, Knoxville, TN, USA; 12 Division of Biological Sciences, University of Missouri, Columbia, MO, USA; 13 Mitrani Department of Desert Ecology, Swiss Institute for Dryland Environmental and Energy Research, Jacob Blaustein Institutes for Desert Research, Ben-Gurion University of the Negev, Midreshet Ben-Gurion, Israel; 14 Department of Environmental Science and Policy, University of California, Davis, CA, USA; 15 Santa Fe Institute, Santa Fe, NM, USA; 16 Africa Program, The Nature Conservancy, Arlington, VA, USA; 17 Institute for Water and Wetland Research, Radboud University, Nijmegen, Netherlands; 18 Physics Department, California Polytechnic State University, San Luis Obispo, CA, USA; 19 Department of Biology, University of Florida, Gainesville, FL, USA; 20 Department of Systematic Zoology, Adam Mickiewicz University, Poznań, Poland; 21 Department of Wildland Resources and Ecology Center, Utah State University, Logan, UT, USA

**Keywords:** Global change, interspecific, intraspecific, long-distance dispersal, population, seed dispersal, spread, variability, within species

## Abstract

As the single opportunity for plants to move, seed dispersal has an important impact on plant fitness, species distributions and patterns of biodiversity. However, models that predict dynamics such as risk of extinction, range shifts and biodiversity loss tend to rely on the mean value of parameters and rarely incorporate realistic dispersal mechanisms. By focusing on the mean population value, variation among individuals or variability caused by complex spatial and temporal dynamics is ignored. This calls for increased efforts to understand individual variation in dispersal and integrate it more explicitly into population and community models involving dispersal. However, the sources, magnitude and outcomes of intraspecific variation in dispersal are poorly characterized, limiting our understanding of the role of dispersal in mediating the dynamics of communities and their response to global change. In this manuscript, we synthesize recent research that examines the sources of individual variation in dispersal and emphasize its implications for plant fitness, populations and communities. We argue that this intraspecific variation in seed dispersal does not simply add noise to systems, but, in fact, alters dispersal processes and patterns with consequences for demography, communities, evolution and response to anthropogenic changes. We conclude with recommendations for moving this field of research forward.

## Teaser

Seed dispersal—the single opportunity for plants to move—is important for plant fitness, species distributions and patterns of biodiversity. Models that predict extinction risk of species, range shifts and biodiversity tend to rely on average dispersal distances. However, we know that seed dispersal is highly variable even within a single species (e.g. some seeds go very far and some barely move away from their parent plant, some seeds end up in great quality habitats and some end up on roads). This paper looks at the consequences of this variation in seed dispersal for plants and their ability to respond to future global change.

## Introduction

For most plants, seed dispersal represents the main opportunity to move and thus has an important impact on plant fitness, species distributions, community composition and patterns of biodiversity (e.g. Merritt *et al.* 2010; Vellend 2010; Kroiss and Hillerslambers 2015). However, models that predict extinction risk of species, range shifts and biodiversity loss rarely incorporate realistic dispersal mechanisms or distances, and tend to assume either global or no dispersal (e.g. Engler *et al.* 2011; Bateman *et al.* 2013). When these models include a more realistic representation of seed dispersal, they tend to rely on mean estimates of dispersal that are assumed to be identical across individuals within a species (e.g. Miller and McGill 2018). By focusing on mean population (or species) estimates, variation among individuals or variability caused by complex spatial and temporal dynamics is ignored. This variation can lead to differences in their seed dispersal effectiveness (*sensu*Schupp *et al.* 2010) as well as in their contributions to long-distance dispersal (e.g. Jordano 2017) and gene flow (Saastamoinen *et al.* 2018). These differences can have important consequences for our ability to understand and predict plant population dynamics, local to regional biogeographic patterns of species and communities, and ecosystem processes.

Individual variation in the seed dispersal process is multifaceted and can include differences in the number of seeds dispersed (e.g. Jordano and Schupp 2000), the specific traits of the dispersed seeds (Wang and Ives 2017), the treatment of the seed during transit (Traveset *et al.* 2007), the dispersal distance (e.g. Thiede and Augspurger 1996) and the quality of the habitat in which they are deposited (i.e. as described by the seedscape, *sensu*Beckman and Rogers 2013). The causes of individual variation in dispersal includes both intrinsic traits of plants (e.g. differences in seed crop size, fruit or seed size, plant height, etc.) and extrinsic characteristics of the environment (e.g. fruiting neighbourhood, habitat structure, community of seed-dispersing animals; Schupp *et al.* 2019). It is also important to recognize that many plant traits affecting seed dispersal, such as fruit diameter, vary not only among individuals, but also within individuals and across years (González-Varo and Traveset 2016; Herrera 2017). Variability in seed dispersal is well documented and is present regardless of the seed dispersal mechanism (see section Seed Dispersal is Influenced by Intrinsic and Extrinsic Variability below, and Schupp, this issue). However, the magnitudes and consequences of intraspecific variation in seed dispersal are poorly understood. We use the term *intraspecific variability* throughout to capture both inter- and intra-individual variability in the dispersal of seeds within species. We acknowledge that the consequences of such variations may sometimes diverge, especially with respect to the evolution of dispersal, but would generally be similar, regardless of the source of variation.

We propose that intraspecific variation in seed dispersal has important implications for our understanding of plant fitness, as well as population, community and landscape dynamics. This is because dispersal estimates based on population means are not the same as dispersal estimates that consider individual variation (Box 1 and Box 2). Chesson has called this effect non-linear averaging (Chesson 1996), and it is based on the mathematical fact that a non-linear function evaluated at its average input values does not yield the same result as evaluating the function over a distribution of input values and then averaging its conditional results. Jensen’s inequality for convex and concave functions is a specific example of non-linear averaging. For example, if the number of seeds produced by an individual is a concave function of its biomass, then Jensen’s inequality (Jensen 1906) implies that variation among individuals in biomass would reduce the population-level mean seed production compared to seed production predicted from mean biomass. Alternatively, if the mean dispersal distance is a convex function with plant height, then variation among individuals in plant height would increase the population-level mean dispersal distance compared to dispersal distances predicted from mean plant height. In stochastic simulations, models based on species- or population-level average dispersal kernels may yield results that are systematically but unpredictably biased in terms of direction and magnitude. Conservation and management efforts require accurate predictions for how species may respond under different management and global change scenarios. With sufficient time, even small systematic biases that may arise by ignoring variation in dispersal have the potential to compound into large misrepresentations.

Box 1. Intraspecific variation in dispersal and non-linear averaging.

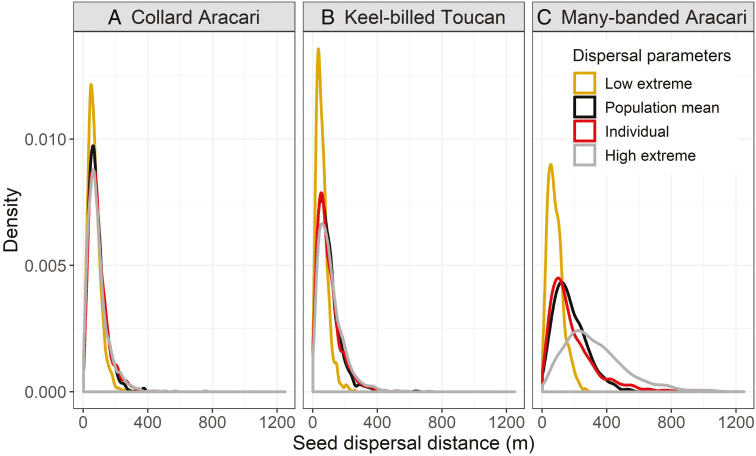

Seed dispersal distances from parent trees for three species of toucans (Ramphastidae) in the New World tropics, A) collared aracari (*Pteroglossus torquatus*), B) keel-billed toucan (*Ramphastos sulfuratus*) and C) many-banded aracari (*Pteroglossus pluricinctus*). Dispersal kernels were generated by combining animal movements from 12 to 23 radio-tracked birds and gut retention times (for *Virola koschnyi*; A, B, Jones (2017) and *Virola flexuosa* seeds; C, Holbrook and Loiselle (2007, 2009)). Gut retention time was based on trials with captive birds, so a single distribution was used with the average gut retention time as the mean in a gamma distribution. We used an exponential distribution to simulate animal movement using four scenarios (low extreme, high extreme, population, individual) and then combined those with simulated gut retention times. For animal movement, exponential distributions were fitted to the radio-tracked individual with the lowest average movement (low extreme) and the individual with the highest average movement (high extreme) to illustrate the range in individual variation in movement. The population-level kernel (population) was fitted using movement data pooled from all individual birds, and the individual-level kernel (individual) used data from each individual separately (i.e. each individual had their own fitted distribution to movement prior to combining across individuals). These results highlight the differences in seed dispersal distances generated by variation in animal movement. They also demonstrate that dispersal kernels generated from the mean population data are not the same as those created from individual kernels. In this particular example, the population kernel underestimates the number of long-distance dispersal (LDD) events (the tail of the curve). For example, with *V. flexuosa* trees in the Ecuadorian Amazon, we defined LDD events as those where seeds were deposited >500 m from their origin by many-banded aracari. The percentage of LDD was 0.6 % under the population-level model, compared to 3.9 % using data that incorporated variation in movement among individuals. For Costa Rican Ramphastids, we defined LDD events as those >200 m. The results were similar, with the population model underestimating LDD events compared to the individual-based model (collared aracari—3.7 % with population model, 6.8 % with individual model; keel-billed toucan—7.3 % with population model, 9.6 % with individual model). Data are available as Supporting Information - Appendix S2.

Box 2. Intraspecific variation in dispersal and kurtosis.

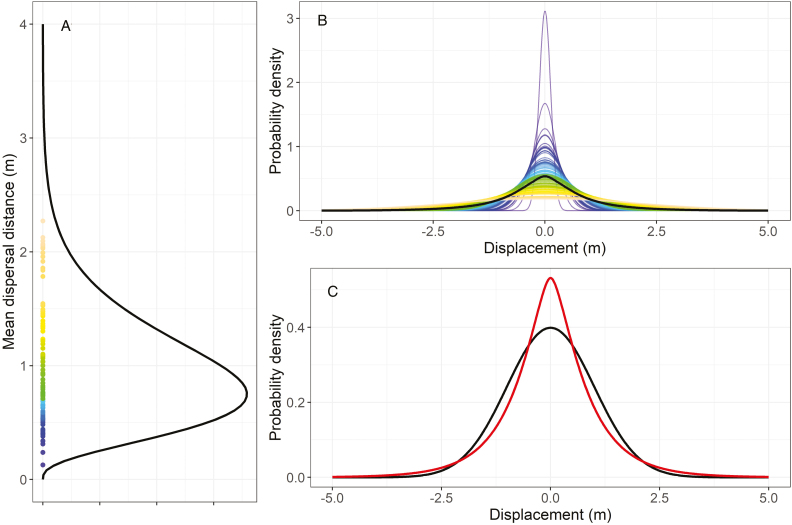

To illustrate that variability in mean dispersal distance creates more leptokurtic dispersal kernels, we can imagine a population of individuals (seeds) each of which exhibits a diffusive movement but whose mean dispersal distance (as determined by their individual diffusion constant) vary. In (A), the distribution among individual mean dispersal distances is gamma distributed with a subsampling of 200 individuals from this distribution shown as coloured points (shape parameter (*s*) = 4, scale parameter (*a*) = 0.25, mean dispersal distance = *s* * *a* = 1 m). In (B), the Gaussian dispersal kernels for each of these 200 individuals are shown using the same colours, and the Gaussian dispersal kernel of the ‘average’ individual (i.e. assuming the mean dispersal distance of 1 m from the gamma distribution) is shown in black. In (C), the population-level dispersal kernel (red) of the heterogenous population is more leptokurtic than the dispersal kernel of a homogenous population where all individuals have the same mean dispersal distance (black). The population-level dispersal kernel *k* is calculated by conditioning, i.e. $k(v)=\int_{0}^{\infty }k_{norm}\frac{v}{L}/Lp(L)dL\;$ where *k*_*norm*_ is the density of a standard Gaussian and *p(L)* is the density of a gamma distribution. Increasing the amount of individual variation leads to more leptokurtic dispersal kernels [see Supporting Information—Appendix S1]. Simulation data are available as Supporting Information - Appendix S3.

In this manuscript, we synthesize recent research that examines intraspecific variation in seed dispersal and its implications for plant ecology to evaluate our current understanding and to recommend avenues for future research to fill remaining knowledge gaps. First, we present a brief overview of how seed dispersal is influenced by intrinsic and extrinsic variability; for more thorough reviews see Schupp (this issue), Côrtes and Uriarte (2012), McConkey *et al.* (2012), Beckman and Rogers (2013), Zwolak (2018). We do not discuss what causes rapid changes in trait variability in plants in any detail, but instead refer interested readers to Johnson *et al.* (2019). Then, we discuss the consequences of intraspecific variation in seed dispersal for local population dynamics, spatial spread, community structure and dynamics, and evolution, and argue that this intraspecific variation in dispersal is not simply adding noise, but altering dispersal processes and patterns. To conclude, we discuss intraspecific variation in seed dispersal within the context of anthropogenic global change and suggest directions for future research.

## Seed Dispersal Is Influenced by Intrinsic and Extrinsic Variability

Intrinsic and extrinsic factors influence the seed dispersal process and variability in these factors contributes to intraspecific variability in dispersal (Box 1, see Schupp, this issue, for a detailed review). Approximately one quarter of trait variability within plant communities exists within species (i.e. morphological and physiological traits; Siefert *et al.* 2015). We highlight intraspecific variability in four types of traits that are known to underlie intraspecific variability in dispersal: fruit and seed size, fruit and seed crop size, plant height and dispersal-specific structures. We also briefly introduce several extrinsic factors that can cause intraspecific variation in dispersal. Variation in these intrinsic and extrinsic factors potentially has significant consequences for plant demography and community composition through its impacts on number of seeds dispersed, the seedscape in which seeds land and the frequency of long-distance dispersal events.

### Fruit and seed size

Fruit and seed size are highly variable both within and among individual plants (Michaels *et al.* 1988), and this variability influences seed dispersal in a variety of ways. For abiotic dispersal, size influences dispersal distance as smaller seeds are typically dispersed further by water (e.g. Delefosse *et al.* 2016) and wind (e.g. Skarpaas *et al.* 2011). For endozoochorous and synzoochorous (where animals intentionally transport seeds without ingestion) dispersers, variation in fruit diameter and seed size can affect how many and which disperser species are able to feed on an individual plant (Galetti *et al.* 2013; González-Varo and Traveset 2016), how seeds are processed (e.g. swallowed or regurgitated, Levey 1987; cached or eaten, Jansen *et al.* 2004; Gómez *et al.* 2008) and how far seeds are moved (Muñoz and Bonal 2008). Individual variation in fruit and seed size and individual variation in the traits of the dispersal agents interact to mediate the realized disperser assemblages of each fruits. This interaction in intrinsic and extrinsic variability has consequences for seed dispersal (Zwolak 2018).

### Fecundity

Individual variation in fecundity has important implications for both long-distance dispersal and number of seeds dispersed, particularly in plants with wind or endozoochorous dispersal (Jordano and Schupp 2000; Norghauer *et al.* 2011). For wind-dispersed plants, highly fecund individuals tend to have longer maximum dispersal distances because increasing the number of seeds released increases the probability of some seeds catching rare updrafts that result in long-distance dispersal (Nathan *et al.* 2002; Norghauer *et al.* 2011; Augspurger *et al.* 2017). Similarly, larger crop sizes for endozoochorous dispersal may also increase the probability of rare, long-distance dispersal events by animals (e.g. *Prunus mahaleb* trees, Jordano and Schupp 2000). The consequences of individual variation in fecundity have not, to our knowledge, been explored for other dispersal modes, but are potentially important with any dispersal system since increasing crop size increases the number of dispersal events and thus the probability of a rare long-distance dispersal event.

### Plant height

Plant height explains much of the variation in dispersal distance across plant species (Thomson *et al.* 2011; Tamme *et al.* 2014; Thomson *et al.* 2018). Together with diaspore terminal velocity and seed abscission, seed release height is a key phenotypic driver explaining individual variation in dispersal distances of abiotically dispersed plants (Thiede and Augspurger 1996; Wender *et al.* 2005). For endozoochorous trees, preferential foraging of frugivores at different canopy heights raises the possibility that differences in height may influence the frugivore assemblage to which fruits are exposed (Poulsen *et al.* 2002; Flörchinger *et al.* 2010) and consequently impact dispersal outcomes.

### Dispersal-specific structures

Intraspecific variation in specialized structures that aid in seed dispersal can also cause intraspecific variation in dispersal kernels. In wind-dispersed plants, pappus and wing morphology can affect seed falling velocity (Riba *et al.* 2005; Tabassum and Bonser 2017). The quantity of low-density tissues in water-dispersed fruits and seeds can affect buoyancy, which may affect dispersal distances (Guja *et al.* 2014). For ant-dispersed species, the presence of elaiosomes and the elaiosome/load ratio increased removal rates by ants (Hughes and Westoby 1992). For fleshy-fruited plants, fruits with relatively higher energetic rewards (e.g. pulp to seed ratio or elaisome size) result in higher removal probabilities (Sallabanks 1993; Willson 1994; Mark and Olesen 1996; Stanley and Lill 2002). For fruit dispersed by epizoochory, there is variation in the presence, size and number of appendages that enable mechanical interlocking with animal fur (Gorb and Gorb 2002) or variation in the degree of heterocarpy, in which individual plants produce morphologically distinct diaspores (Monty *et al.* 2016). However, the impact of this variation on dispersal has not yet been tested for this dispersal mode. Intraspecific studies are also rare among synzoochorous species (e.g. Smallwood *et al.* 2001; Shimada *et al.* 2010), although their results consistently suggest that seeds that offer greater rewards and have fewer defences or lower handling times are dispersed further. These same traits also mean that they are consumed at higher rates, with less perishable seeds cached more frequently (reviewed by Vander Wall 2010; Lichti *et al.* 2017).

### Extrinsic factors

Extrinsic factors related to a plant’s local environment and its dispersal vector can cause intraspecific variation in dispersal. For example, the interaction between abiotic dispersal vectors and the landscape structure can cause intraspecific differences in water dispersal due to local flow patterns (Van der Stocken *et al.* 2015) and in wind dispersal due to local topography, atmospheric conditions and surrounding vegetation (Nathan *et al.* 2001; Augspurger *et al.* 2017). Animal-dispersed plant species are impacted by individual variation among seed dispersers (reviewed in Zwolak 2018) and these may interact with intrinsic factors, such as fruit and seed size as discussed above. Animal behaviour and the plants surrounding a focal plant can also interact with the local fruiting neighbourhood impacting dispersal probabilities and distances (Blendinger *et al.* 2008; Carlo and Morales 2008). Finally, differing impacts of anthropogenic drivers across space also causes within-species variation in dispersal; for example, habitat fragmentation can influence frugivore movement patterns and thus dispersal distances (Levey *et al.* 2005) and defaunation impacts the composition of the frugivore assemblage and behaviour of remaining frugivores (McConkey and Drake 2006; Holbrook and Loiselle 2009).

## Consequences for Local Population Dynamics

Intraspecific variation in seed dispersal can affect demography by influencing vital rates (i.e. germination, growth and survival) as well as dynamics within and among populations (Howe and Miriti 2004). For example, variation in seed dispersal distance can lead to variation in plant survival and growth as some seeds may escape mortality due to natural enemies (Janzen 1970) or experience reduced competition from siblings (Cheplick 1992). Variation in how seeds are dispersed can also lead to variation in survival depending on the time and treatment of seeds passing through the gut for endozoochorous species (Traveset *et al.* 2007), and the quality of habitat in which seeds are deposited (Beckman and Rogers 2013). It is critical to recognize the effect of this variation, as these vital rates determine population growth. In addition, individual variation in dispersal can affect metapopulation processes by impacting the frequency of movement, genotypes and traits of individuals that move between populations (Cheptou *et al.* 2008; but see Castorani *et al.* 2017).

Overlooking intraspecific variation in dispersal can impact conclusions of local population persistence in several ways, particularly in changing environments. First, individual variation in dispersal may impact projections by population matrix and integral projection models. In these models of local population dynamics, dispersal is rarely considered explicitly (see next section for discussion of population spread), but instead subsumed into the various factors affecting the transitions from seed to seedling, or seedling to sapling. Because single individuals can contribute large portions of new recruits in plant communities (Wheelwright 1986; Minor and Kobe 2017), estimated population-level recruitment may change substantially as individual composition changes (e.g. between population, or within populations if the so-called super-producers die). In addition, demographic models that do not explicitly consider dispersal are unable to forecast how altered dispersal processes (i.e. due to defaunation, fragmentation or changing climates) may influence population dynamics and persistence (see section Relevance under Anthropogenic and Global Climate Change below). Models that more mechanistically consider how dispersal and the deposition environment impact growth and survival can incorporate these processes and project population trajectories under altered dispersal (e.g. Caughlin *et al.* 2014).

A few phenomenological and mechanistic models do explicitly address how dispersal influences local population dynamics (e.g. Godinez-Alvarez and Jordano 2007; Brodie *et al.* 2009; Loayza and Knight 2010). This is an important first step in understanding the importance of dispersal. Next, researchers should examine how using the mean values related to dispersal (e.g. dispersal distance, fecundity) biases population projections to identify the circumstances when intraspecific variation in dispersal needs to be considered for projecting population dynamics. For example, when estimating dispersal distances based on trait allometries (e.g. Norghauer *et al.* 2011), the use of mean trait values can under- or overestimate dispersal distances due to Jensen’s inequality. In particular in small populations, individual variation in dispersal can cause population-level patterns of dispersal to differ significantly from expectations based on mean values (Lewis and Pacala 2000). Simulations that explicitly include intraspecific variation are not equal to models that use the mean and variability of the population (Box 3), with the largest consequence for populations occupying habitats located far away from sites suitable for establishment. Overall, there is a need for demographic studies to include dispersal explicitly and to explore how and when intraspecific variation in dispersal affects local population dynamics.

Box 3. Intraspecific variation in dispersal and demography.

**Simulation setup:** A 640-m-long wrapped transect (i.e. seeds dispersing off one edge, reappear on the opposite edge). The initial population was restricted to a contiguous 80 m, centred either at 0 (yellow), 80 (grey) or 160 (dark grey). For the ‘Harsh environment’ simulations, half of the transect was made relatively uninhabitable for seedlings (dashed).

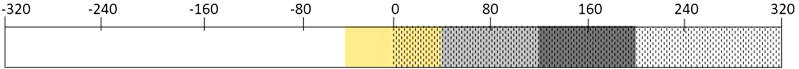

**Simulation results:** In the Uniform environment, the results are the same regardless of the starting location. For simplicity, only one is shown (location = 0).

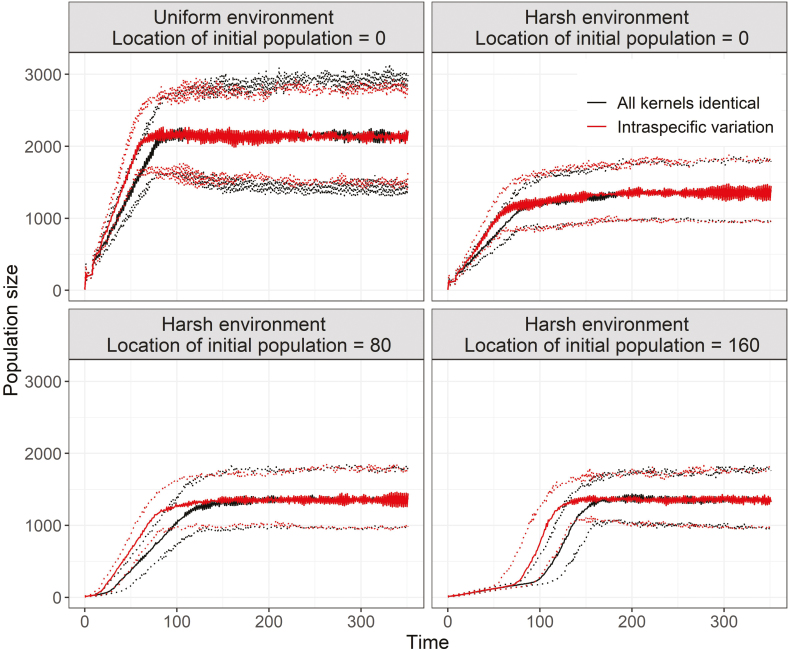

The median (solid lines) and 95 % quantiles (dashed lines) of population size are shown, from *n* = 50 replicate simulations per scenario. All simulations used identical rules for individual growth, fecundity and survival. Seeds were dispersed from individual maternal plants according to Gaussian kernels, with a population-level mean dispersal distance of 144 m. For ‘all kernels identical’ scenarios, this mean dispersal distance was applied to all individuals. For scenarios with ‘intraspecific variation’, 20 % of the plants dispersing seeds in a given year were randomly assigned a mean dispersal distance of 464 m, and the remainder had a mean distance of 64 m. Thus, while the population-level mean dispersal distance was identical in both cases, the intraspecific variation scenario had more plants dispersing seeds to short distances while a few plants are dispersing seeds much further. Seedling establishment probabilities and initial population locations also varied by scenario. All simulations included increasing survival with distance from already established plants; however, in the ‘Harsh environment’ scenario, the upper half of the transect was made uninhabitable to new seedlings (e.g. as might occur under severe browsing pressure). In all cases, initial populations were restricted to a contiguous 80 m segment of the transect; however, this segment was centred either at the middle of the transect (location = 0) or closer to the upper half of the transect (location = 80 m, or 160 m). For the ‘Harsh environment’ scenarios, this resulted in different levels of seed limitation as the source population was increasingly isolated from the lower, more habitable portion of the transect. While the final population size was unaffected (i.e. the asymptote), intraspecific variability in dispersal consistently increased population growth rates at the start of the simulations. The greatest difference in population growth rates was found when the source population was the most isolated from suitable habitat (location = 160). Data are available as Supporting Information-Appendix S4.

## Consequences for Spatial Spread of Populations

Explaining historical range expansions and predicting future vegetation migration rates is a fundamental question in global change biology and invasion ecology (Clark *et al.* 1998; Lockwood *et al.* 2013). As traditional Gaussian dispersal kernels required an unrealistically large mean dispersal distance to match historical spread rates (i.e. ~1000 m, Clark *et al.* 1998), leptokurtic kernels were proposed as an alternative to describe spatial spread (Mollison 1977; Kot *et al.* 1996). In contrast to their Gaussian counterparts, these kernels have higher probabilities of short-distance dispersal events, creating a more peaked distribution, and higher probabilities of rare, long-distance dispersal events, creating a fatter or thicker tail (Box 4). These dispersal kernels preserve the mean distance travelled by a seed, but lead to faster rates of either constant or ever-accelerating spatial spread (Box 4).

Box 4. Rare long dispersal distance events increase rates of spatial spread.

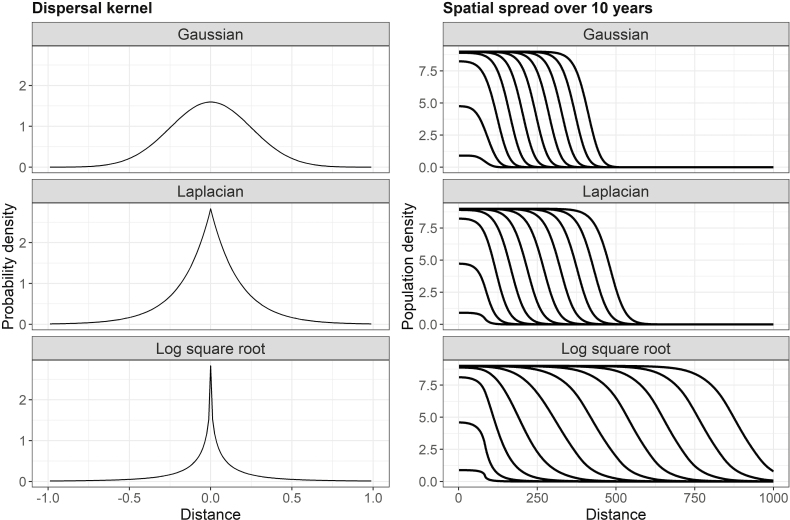

The Gaussian (thin-tailed), Laplacian (leptokurtic) and log square root (leptokurtic and fat-tailed) dispersal kernels plotted on the left-hand side represent the density of seeds dispersed a given distance north or south from its parent. The spatial distribution of the corresponding populations over a 10-year period are shown to the right. Leptokurtic dispersal kernels, such as the Laplacian and log square root, result in faster spread rates than the Gaussian dispersal kernel. Exponentially bounded dispersal kernels, such as the Gaussian and Laplacian, result in asymptotically constant spread rates, whereas distributions that are not exponentially bounded (i.e. fat-tailed, Kot *et al.* 1996), such as the log square root, result in ever-accelerating spread rates. All dispersal kernels have a mean dispersal distance of 0.25 and local survival and reproduction is given by a Berverton–Holt function 10 * *n*/(1 + *n*), where *n* is the local density.

These population-level leptokurtic kernels could arise due to intraspecific variation in seed dispersal (Box 2), and have been the focus of numerous studies (Neubert and Caswell 2000; Petrovskii and Morozov 2009; Bouin *et al.* 2012; Stover *et al.* 2014; Horvitz *et al.* 2015; Schreiber and Beckman, 2019). For example, Neubert and Caswell (2000) studied spatial spread of the Neotropical *Calathea ovandensis* due to dispersal by four ant species. The one ant species, *Pachycondyla apicalis*, that dispersed seeds the furthest (mean dispersal distance > 9 m) only dispersed 7 % of the seeds. However, the inclusion or removal of *P. apicalis* led to a large change in the rate of spatial spread. Horvitz *et al.* (2015) reached a similar conclusion with gravity-, catbird-, robin- and raccoon-dispersed seeds in *Ardisia elliptica*. Using frugivore-stratified spread models, they showed that it was the infrequent but longer-distance dispersal by robins that determined rates of spatial spread. As illustrated in Box 2, continuous variation in the diffusion coefficient *D* (i.e. the square root of the mean squared displacement) among individuals can generate leptokurtic dispersal kernels at the population level and, thereby, increase rates of spatial spread (Petrovskii and Morozov 2009; Stover *et al.* 2014; Schreiber and Beckman, 2019). The magnitude of this increase, however, depends subtly on the nature of the distribution underlying the individual variation. For example, gamma-distributed individual variation in *D* leads to fatter exponentially bounded population-level dispersal kernels (Petrovskii and Morozov 2009). Dispersal kernels that are exponentially bounded result in asymptotically constant spread rates (Box 4). As a first-order approximation, the increase in rate of spatial spread due to individual variation in *D* is proportional to the variance of this variance (Stover *et al.* 2014; Schreiber and Beckman, 2019). In contrast, individual variation in *D* could lead to a population-level dispersal kernel with a power-law tail. As these power-law tails are not exponentially bounded, this form of individual variation would lead to ever-accelerating rates of spatial spread (Box 4; Kot *et al.* 1996). An example of a process that could lead to a population-level dispersal kernel with a power-law tail arises from individual variation in body size of animal seed dispersers that may affect the movement of seeds—seed dispersers with higher body mass are expected to change movement direction less frequently than seed dispersers with lower body size as it requires a larger force and larger energy expense (Petrovskii and Morozov 2009).

Spread rates are determined by both dispersal and demography at or near the front of an invasion. To understand how individual variation in dispersal affects spread, one must simultaneously assess the role of variation in demography (introduced in the previous section). Variation in dispersal and demography can have a common, external source, which then influences spread through both pathways. In a study in which individual variation was induced externally by a receptacle-feeding weevil that forms cysts inside developing flower head of *Carduus nutans*, Marchetto *et al.* (2014) showed that cysts affect the number of seeds produced, the probability that a seed would be dispersed away from the mother plant and the terminal velocity of seeds that do disperse. The consequences for population growth and spread were evaluated with matrix-based travelling wave-speed models (e.g. Neubert and Caswell 2000): the average load of cysts had a greater effect on population spread than local population dynamics of *C. nutans* in New Zealand (−43 % vs. −17 %), but the reverse was true for the USA (−46 % vs. −64 %). This example further shows that population-level impacts depend not just on the amount of variation in dispersal-related seed traits, but also on the sensitivity of the spread rate to changes in those traits. That sensitivity is jointly shaped by all dispersal, reproduction, survival and growth rates that co-determine the life cycle. While the weevils in the above case study have clear negative effects on both dispersal and fecundity, there may be situations where covariance of dispersal and demography rates can propel population spread. When individual variation in fecundity is positively correlated with mean dispersal distance (e.g. a higher probability of long-distance dispersal for seeds of more fecund parents), this can substantially increase spread rates, as shown in theoretical explorations (Schreiber and Beckman, 2019).

The aforementioned theoretical insights about the effects of individual variation on dispersal are based on the assumption that the populations are experiencing negative density dependence at the population edge. Thus, it is the dispersal traits of the individuals at the leading edge that are ‘pulling’ the population forward. Alternatively, there can be a strong Allee effect at the leading edge, which is a positive density-dependent process. When this occurs, it is the individuals dispersing from the core to the leading edge that are important (i.e. the population is being ‘pushed’ forward). Self-incompatible plants are most likely to experience Allee effects due to mate limitation, thus a potentially important challenge for future work is understanding how individual variation in seed dispersal influences the initiation of spatial spread and the rate of spatial spread. These theoretical insights have also assumed that environmental conditions are relatively homogenous in time and space. While a useful first-order approximation, environmental variation can substantially alter rates of spatial spread (Shigesada and Kawasaki 1997; Ellner and Schreiber 2012). For example, spatial variation in fecundity can reduce spread rates (Shigesada *et al.* 1986), while temporal variation in dispersal rates can increase spread rates (Ellner and Schreiber 2012). Hence, another important challenge for future work is to identify how the joint effects of environmental and seed dispersal variation impact rates of spatial spread.

## Consequences for Plant Communities

Dispersal is integral to local community dynamics, as species arrival depends on successful dispersal and establishment from the local and regional species pools (Leibold *et al.* 2004; Holyoak *et al.* 2005). Thus, intraspecific variability in dispersal (e.g. variability in dispersal distance, quantity or quality of dispersal, or species composition of the incoming propagules) has the potential to influence community processes such as assembly, composition and species coexistence. To the best of our knowledge, there have been no experiments that directly examine intraspecific variability in dispersal at the community level. However, significant variability in dispersal at the population level across time or space can indeed alter community dynamics, indicating that intraspecific variation in dispersal should affect community dynamics. For example, plant species richness increases with the richness of species’ propagules that arrive (Tilman 1997; Aicher *et al.* 2011), as well as with the distance from which these species arrive (Germain *et al.* 2017). Therefore, we can hypothesize that intraspecific variability in dispersal distance and quantity could alter the species richness of a community. Additionally, intraspecific variation in the timing of species arrival can alter community assembly, as some species have strong priority effects that alter overall diversity if they arrive first (e.g. Fukami *et al.* 2005; Martin and Wilsey 2012).

In theoretical systems, stable coexistence can also be impacted by intraspecific variation in dispersal. High variation among individuals (Clark 2010), or variation in the environmental conditions that in turn create individual variability (Berkley *et al.* 2010), may promote coexistence through niche partitioning in many dimensions. This individual-level variation can also make coexistence more difficult as it can increase the dominance of superior competitors, reduce species-level niche differentiation and increase the effects of demographic stochasticity (Hart *et al.* 2016). Currently, we have a limited understanding of the importance of intraspecific variation in dispersal for plant communities. Intraspecific variation could matter for community-level processes (e.g. timing of arrival, diversity of arrival, priority of arrival, etc.) but little is known empirically or theoretically of the consequences for diversity. However, individual-based models and other approaches make these kinds of studies possible. Increased variation in traits can increase or decrease coexistence (Clark 2010; Hart *et al.* 2016), but studies to date have not explicitly incorporated intraspecific variation in dispersal. The hypotheses outlined here offer a rich area for future studies to test.

## Consequences for Evolution

Seed dispersal and pollen dispersal are the primary sources of gene flow in plants. As such, seed dispersal plays a central role in evolutionary biology, driving patterns ranging from population structure (Hamrick and Godt 1996) to inbreeding depression (Roze and Rousset 2005) and local adaptation (Tigano and Friesen 2016). Moreover, the high level of intraspecific variation in seed dispersal provides the foundation for the great evolutionary potential of dispersal itself (Ronce 2007). Natural selection can act on this variation because two conditions are generally fulfilled. First, seed dispersal has a genetic basis with heritability estimates of up to 0.8 (Saastamoinen *et al.* 2018), although heritability strongly depends on the environment (Donohue *et al.* 2005; Wender *et al.* 2005). Second, all elements of dispersal as discussed here can strongly influence maternal fitness. The number of seeds produced determines the potential number of offspring, while subsequent offspring survival and reproductive success depend on where seeds land within the seedscape (Rubio de Casas *et al.* 2012). Evolution may reduce or maintain intraspecific variation in dispersal. Specific dispersal phenotypes—that either promote or limit dispersal—may be lost if they are selected against (e.g. Cheptou *et al.* 2008) or through genetic drift (Barrett and Kohn 1991). However, because the evolutionary costs and benefits of dispersal highly depend on environmental conditions and the maternal phenotype (Ronce 2007), contrasting selection pressures in heterogeneous environments can maintain dispersal variation within populations (Mathias *et al.* 2001; Stevens *et al.* 2010). Evolution may also promote variation within the seed crop of an individual in temporally variable environments (Snyder 2011; Rubio de Casas *et al.* 2012). Heterocarpy, where individuals produce fruits with multiple dispersal morphologies, is especially common in the Asteraceae and Chenopodiaceae (Imbert 2002), and has been shown to have a certain degree of phenotypic plasticity (Taghizadeh *et al.* 2009; Rubio de Casas *et al.* 2012).

In spreading plant populations, heritable individual variation in dispersal can lead to spatial sorting, a fitness-independent process where highly dispersive genotypes accumulate and reproduce at the leading edge (Shine *et al.* 2011; Bouin *et al.* 2012). This results in the spread process itself selecting for the very traits that promote increased dispersal (Travis and Dytham 2002; Perkins *et al.* 2013) and accelerates the rate of spatial spread (Phillips *et al.* 2010; Bouin *et al.* 2012). Williams *et al.* (2016) showed that populations of *Arabidopsis thaliana* invading experimental landscapes evolved higher dispersal abilities at the invasion front, and that evolution accelerates the spread velocity up to 200 % in fragmented landscapes. However, mixed evidence for evolutionary change in seed dispersal has been found for range expansions under field conditions (e.g. Bartle *et al.* 2013; Huang *et al.* 2015; Monty *et al.* 2016), which require further study.

Individual variation in dispersal strongly affects maternal fitness, facilitating rapid evolution of dispersal and associated traits with consequences for populations and communities as discussed in the previous sections. Focusing on the mean dispersal value of a population will ignore key standing genetic variation around this mean, which will determine the probability of an adaptive allele reaching fixation and whether the population can rapidly evolve increased or decreased dispersal in the future. The resulting eco-evolutionary dynamics are a major driver of plant population responses to rapid environmental change.

## Relevance under Anthropogenic and Global Climate Change

Ongoing and future climate change will lead to increasing temperatures, changes in precipitation regimes, and an increase in the frequency and intensity of extreme events (i.e. drought, floods, heat waves, etc.) (IPCC 2014). This increase in extreme weather events may have a direct effect on the frequency of long-distance dispersal events, in particular for areas affected by hurricanes and storms (Gillespie *et al.* 2012). In response to changes in climate, species may shift their distributions to stay within their bioclimatic niche, adapt to the new environmental conditions or become locally extinct (Dawson *et al.* 2011; Hof *et al.* 2011). As species shift their ranges, changes in community composition (Lloret *et al.* 2009) and even potentially novel communities (Williams and Jackson 2007) are likely, which can alter ecosystem functioning (e.g. Liu *et al.* 2018; Morin *et al.* 2018). Thus, seed dispersal will play a critical role for how plants and ecosystems respond to climate change and there may be additional consequences of ignoring intraspecific variation within this global change context.

As discussed above, ignoring intraspecific variation in seed dispersal can underestimate population spread rates. This is critical for predicting future range shifts, as the ability of plants to track rapid changes in climate remains largely uncertain (e.g. Zhu *et al.* 2012; Cunze *et al.* 2013). By not considering dispersal variation, previous estimates may have systematically underestimated plant migration rates (see Box 2; individual variation creates more leptokurtic dispersal kernels at the population level leading to more long-distance dispersal). However, it is important to note that even with faster migration rates, many plant species will still be unable to reach new bioclimatically suitable habitat due to landscape fragmentation and life history constraints (Miller and McGill 2018). While fragmentation can select for increased dispersal ability during population spread (Williams *et al.* 2016), severe fragmentation may select for reduced dispersal capabilities in metapopulations (Cheptou *et al.* 2008), further limiting plants’ abilities to persist in fragmented landscapes. Whether dispersal is constrained by life history traits within plant species is an open question. Bonte and Dahirel (2017) propose that dispersal traits evolve independently from other life history traits, but studies for plants documenting intraspecific variation in dispersal in the context of life history strategies are limited. By incorporating intraspecific variability in seed dispersal, it will increase our ability to predict the vulnerability of species to decline or even local extinction (Valladares *et al.* 2014; Cochrane *et al.* 2015) and will help inform alternative conservation efforts, such as assisted dispersal (Hallfors *et al.* 2017). Climate change may also have a direct influence on seed production and seed traits. For example, increasing temperatures reduced both seed set and seed size in kidney beans (*Phaseolus vulgaris*) (Prasad *et al.* 2002). As discussed above, variability in seed crop size and seed size affects the probability of seeds reaching favourable habitats which may ultimately alter community composition and ecosystem functioning. To be clear, plants are expected to face challenges in keeping pace with rapid climate change, and even the most optimistic scenarios assume their dispersal kernels remain the same. However, if climate change also alters seed traits (as it appears to), then this induces an additional barrier or benefit for successful seed dispersal.

Climate change is not the only threat that species have to cope with. Anthropogenic activities, such as fragmentation, hunting of animal seed dispersers and harvesting of plants, can also be strong selective forces. Anthropogenic activities have already been shown to alter plant traits and the amount of variation between individuals in plant populations (Hall *et al.* 2003; Law and Salick 2005; Galetti *et al.* 2013) as well as disrupting the co-evolutionary dynamics of plant–frugivore interactions, leading to novel selection pressures on dispersal-related seed traits (Fontúrbel and Medel 2017). For example, human harvesting of larger individuals has resulted in a systematic reduction in plant height of a rare plant species (*Saussurea laniceps*) (Law and Salick 2005). In addition, habitat loss and hunting have contributed to the functional loss of large seed dispersers, resulting in a reduction in seed size in a tropical palm population (*Euterpe edulis*) (Galetti *et al.* 2013). This anthropogenically induced reduction of intraspecific variation is often the opposite direction from that which would be driven by natural selection (Carlson *et al.* 2007). The loss of individual variation may reduce fecundity and recruitment and have consequences under ongoing climate change (Law and Salick 2005; Galetti *et al.* 2013). Moreover, the reduction of seed size in some plant species may cause increased vulnerability as smaller seeds are more sensitive to desiccation (Galetti *et al.* 2013; Wyse and Dickie 2017), which will be especially relevant during extended and intensified periods of drought under future climate change. Finally, individual variation in dispersal distance may affect the ability of populations to adapt to these anthropogenic changes, by altering the spatial scale of gene flow. Although gene flow can provide a source of adaptive genetic variation, it may also homogenize populations and prevent local adaptation (reviewed in Tigano and Friesen 2016). The ability to adapt to novel climate conditions or biotic interactions will be critical to the persistence of many populations under rapid environmental change (Gonzalez *et al.* 2013).

The eco-evolutionary dynamics of dispersal will play a key role for determining species responses to habitat fragmentation, biological invasions and range shifts in response to climate change (Travis *et al.* 2013; Urban *et al.* 2016). As noted in previous sections, dispersal-related traits may rapidly evolve during population spread through favourable habitat. However, these evolutionary changes may affect how populations subsequently respond to stressful environments such as those expected during range shifts under climate change. In experimental invasions of *A. thaliana*, an evolutionary increase in seed size over six generations of spread was associated with a subsequent reduction in population performance under drought stress (Lustenhouwer *et al.* 2019). Thus, intraspecific variation in dispersal and seed traits and their evolution will influence the ability of plants to respond to anthropogenic and global climate change.

## Recommendations for Best Practices and New Approaches for Studying Individual Variation and Its Implications through Combining Empirical and Modelling Studies

As demonstrated throughout this manuscript, ignoring intraspecific variation in dispersal can have important consequences for our understanding of population and community dynamics, spatial spread and evolution, and is especially relevant under future global changes. Below we outline some recommendations that are intended to advance this field of research.

First, a paradigm shift is necessary regarding the way we think about dispersal. The default, *a priori* assumption should be that intraspecific variation in dispersal exists and is biologically relevant. With this in mind, reporting of mean dispersal distance *plus* some measure of variance (i.e. standard deviation, variance, range) should become standard practice in order to begin quantifying uncertainty due to intraspecific variation. Statistical approaches, such as hierarchical Bayes models, can allow researchers to quantify intraspecific variability arising from different sources (Albert *et al.* 2011; Nuñez *et al.* 2019). Due to the disproportionate amount of influence that rare events can cause, explicitly noting the presence of outliers is also important. As the information on fruit and seed traits accessible from publicly available databases continues to grow (TRY Plant Trait Database (Kattge *et al.* 2011); KEW Seed Information Database (http://data.kew.org/sid/); LEDA (http://www.uni-oldenburg.de/en/biology/landeco/research/projects/leda); FRUBASE (https://doi.org/10.5061/dryad.9tb73)), we recommend researchers make dispersal distance data available along with information on traits, dispersers and environmental context (e.g. (e.g. Tamme *et al.* 2014; Sullivan *et al.* 2018). One of the aims of the CoDisperse Network is to create such a centralized dispersal database along with developing standardized protocols to ensure the necessary data are available for simultaneously parameterizing and developing models, and testing model predictions (Beckman *et al.*, 2019).

Second, models that describe the responses of populations, communities or ecosystems need to explicitly account for this variation. This is not a simple task and will require the diverse perspectives of mathematical, computational and statistical ecologists to develop a variety of approaches. We first need to understand how the number of seeds dispersed and the resulting spatial patterns change as a function of parental phenotype, seed phenotype and environmental context (as well as how context changes over space and time). One can take a bottom-up, mechanistic approach for modelling seed dispersal (e.g. Nathan *et al.* 2011; Côrtes and Uriarte 2012), or one can use a top-down, phenomenological approach by directly fitting dispersal kernels to field data (e.g. Lustenhouwer *et al.* 2017) as applied to interspecific variation in dispersal. One suggestion would be to sample across the variable space for factors that are known to affect dispersal, ensuring sufficient sampling to estimate a kernel at each combination of predictor values (Box 5).

Box 5. Developing dispersal kernels that include intraspecific variation.

Averaging over either space or time essentially destroys all rare or location-dependent events that cause long-distance dispersal or location-specific effects (e.g. wind gusts, sharp hill inclines, location-dependent top wind speeds or wind directions, etc.), often resulting in severe underestimation of dispersal distances and significant smoothing of the total distribution. Instead, we can integrate (average) over phenotype heterogeneity instead of space or time. One possibility is to form a composite seed shadow by segmenting space (and if necessary, time) into a raster grid, mapping any conditions that change over space or time (e.g. variable wind fields, parent height), calculating dispersal from each pixel to the other pixels conditional on local conditions and then summing arrivals for each pixel. In doing so, care must be taken to integrate over both the source and destination pixels, as well as any traits or conditions that do not vary over space and time. One must also be aware that the creation of these pixels from the underlying discrete data set is a process of averaging itself. If the pixel grid is overly coarse, this averaging will have all of the same consequences mentioned above. Provided that the fates of individual seeds are conditionally independent (i.e. the movement of one seed provides no information on the movement of other seeds after accounting for the effects of environmental variables) and identical (any two seeds with the same traits that are subject to the same conditions can be exchanged with each other), boa population-level dispersal kernel can be derived by summing the dispersal kernels associated with each combination of traits and environmental conditions, weighted by their probability of occurrence in the population as a whole. There is one important caveat to this approach: some of the phenotypic traits that influence dispersal also influence germination, seedling survival and growth (e.g. seed size; Skarpaas *et al.* 2011). If the goal is to model population or community processes or trait evolution, we cannot average over traits because we will then lose track of them and their downstream effects.

Both approaches have their strengths and limitations. The phenomenological approach could be a valuable tool to compare the magnitude of variation in dispersal within sets of individuals in various environments. On the other hand, predictions based on top-down statistical approaches are liable to fail when confronted with novel conditions that fall outside the domain of the original data, such as those expected from global change. Mechanistic models would be better suited for simulating population to ecosystem responses under specific future conditions or scenarios that would be difficult to sample. The availability of appropriate data present challenges for both types of approaches, and can limit our ability to choose which variables or processes are important to incorporate during model development (Urban *et al.* 2016; Lustenhouwer *et al.* 2017; Beckman *et al.*, 2019). Other relevant, interdisciplinary approaches are discussed by Rogers *et al.* (this issue) in the context of describing total dispersal kernels and Johnson *et al.* (this issue) in the context of rapid changes in dispersal.

These updated dispersal kernels then can be utilized in a variety of population and community models to explore the consequences of intraspecific variation of dispersal for plant populations and communities. For an overview of how these dispersal kernels can be integrated with population models, see Beckman *et al.* (2019) and Jongejans *et al.* (2008). While the community-level consequences of intraspecific variation in dispersal may be difficult to study in the field, simulation models that integrate empirical studies, evolutionary perspectives and theory can provide a predictive understanding of plant communities and ecosystems in response to variability in dispersal. For example, dynamic vegetation models (DVMs) range from individual-based models that simulate community dynamics using species-specific parameters for establishment, growth, competition and mortality to cohort-based models that simulate biogeochemical cycles and vegetation distributions using plant functional types (Snell *et al.* 2014). Dynamic vegetation models incorporate information on plant demography, physiology, and simulate interspecific competition for light, water and nutrients (e.g. SORTIE (Pacala *et al.* 1996), FORMIND (Köhler and Huth 1998), TreeMig (Lischke and Löffler 2006; Meier *et al.* 2012)), but require mathematical, computational and empirical advances to represent the spatial and temporal scales relevant for seed dispersal (Snell *et al.* 2014). Although DVMs have not yet been used to simulate intraspecific variability in seed dispersal, this would be a promising research approach to explore. More specifically, significant advancement could be made by performing targeted empirical experiments to determine how various global change factors will alter distributions of dispersal traits, and then incorporate these validated distributions into the above-mentioned individual-based models.

Although we have presented these recommendations separately, we envision an iterative cycle of model building and data collection. Quantitative and empirical ecologists would collaborate to identify the important drivers of individual variation in different dispersal syndromes, characterize their distributions and validate model predictions. Ideally, in the long-term as capabilities increase, studies should characterize not only the marginal variation in drivers, but how those variables change with regard to underlying environmental drivers, genetics, ontology and phenology, and should also characterize covariation in relevant seed or parental traits. There are many exciting research directions that can be pursued with the use of field (e.g. observational, experimental) studies, modelling studies (e.g. statistical, computational, mathematical) and their interface.

## Conclusions

We demonstrate that individual variation in seed dispersal is important to consider for responses of populations and communities, especially under global change scenarios. Future studies on intraspecific variation in dispersal are recommended to further elucidate how the dynamics of populations, communities and evolution are affected. More specifically, we suggest (i) measuring and reporting variability in seed dispersal to quantify variance, (ii) incorporating variability in dispersal into models to simulate its effect and (iii) using the results of the models to design experiments to test the predictions about the role of intraspecific variability in seed dispersal.

## Supplementary Material

plz016_suppl_Supplementary_Appendix_S1

plz016_suppl_Supplementary_Appendix_S2

plz016_suppl_Supplementary_Appendix_S3

plz016_suppl_Supplementary_Appendix_S4

## Supporting Information

The following additional information is available in the online version of this article—

**Appendix S1.** Leptokurtic kernels with different amounts of variation.

**Appendix S2.** Toucan data used for Box 1.

**Appendix S3.** Simulation data used for Box 2.

**Appendix S4.** R code for simulating demographic data shown in Box 3.
