# Supplementary material for: Consequences of intraspecific variation in seed dispersal for plant demography, communities, evolution and global change
Source: AoB Plants. 2019 Mar 21;11(4):plz016. doi: 10.1093/aobpla/plz016 (PMC6644487; doi:10.1093/aobpla/plz016)
Supplement: plz016_suppl_Supplementary_Appendix_S1 [file plz016_suppl_supplementary_appendix_s1.docx]

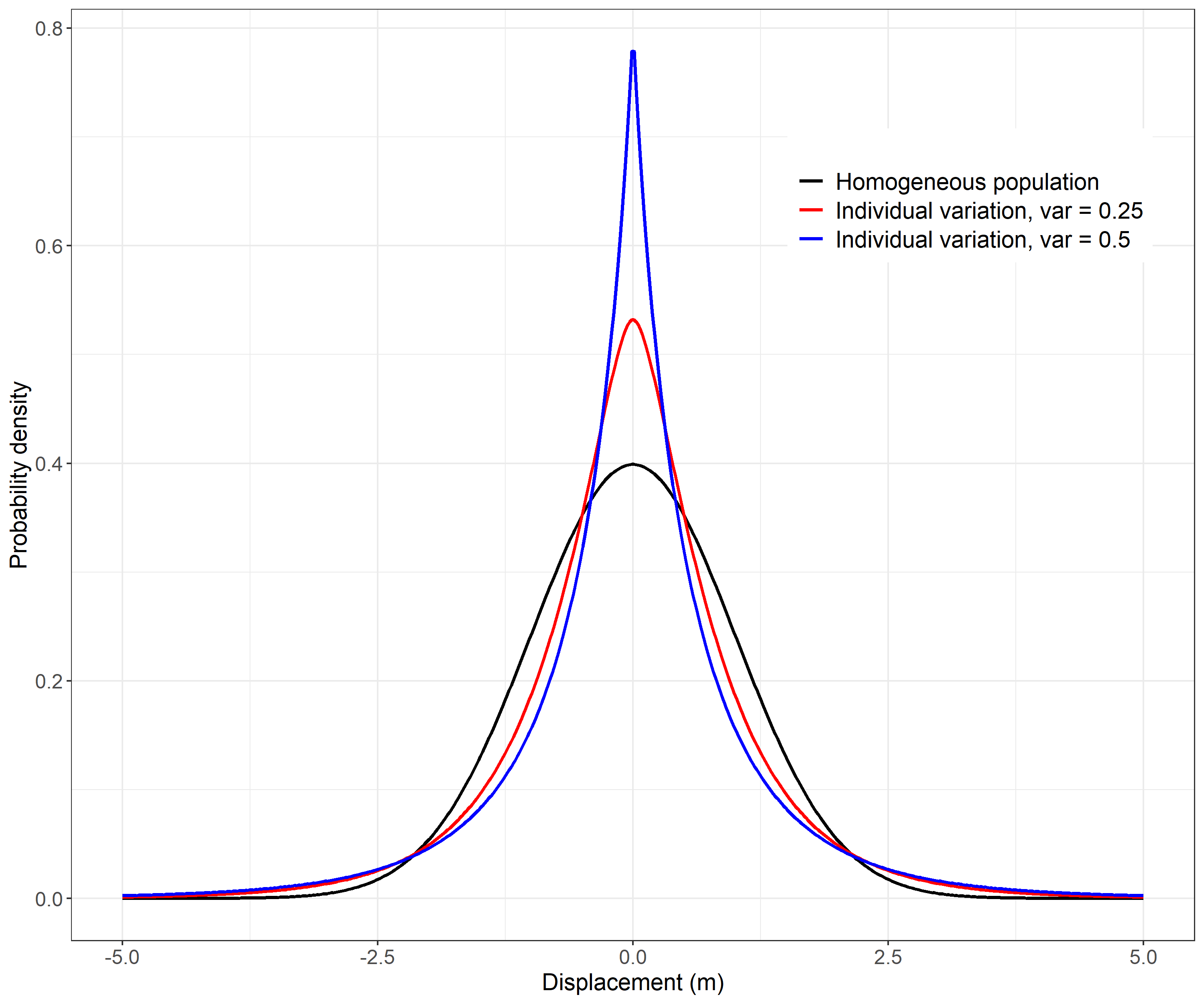


**Appendix 1.**

**Increasing variation, leads to more leptokurtic kernels.** Shown are the population level dispersal kernels (see Box 2 for additional information). In black is the dispersal kernel of homogeneous population (i.e. all individuals had the same mean dispersal distance of 1 m). In red is the dispersal kernel for the heterogeneous population with a variance of 0.25 (shape parameter = 2, scale parameter = 0.25, mean dispersal distance = 1), and in blue is the dispersal kernel for another heterogeneous population with increased variance of 0.5 (shape parameter is 1, scale parameter = 0.50, mean dispersal distance = 1).
